# Effect of ethyl acetate extract of usnea longissima on esophagogastric adenocarcinoma in rats^1^

**DOI:** 10.1590/s0102-865020190030000005

**Published:** 2019-03-18

**Authors:** Renad Mammadov, Bahadir Suleyman, Durdu Altuner, Elif Demirci, Nihal Cetin, Adnan Yilmaz, Huseyin Baykal, Hilal Alpcan, Emine Akyuz Turumtay, Halis Suleyman

**Affiliations:** I Assistant Professor, Department of Pharmacology, Faculty of Medicine, Erzincan Binali Yildirim University, Erzincan, Turkey. Scientific, intellectual, conception and design of the study; manuscript preparation.; II Assistant Professor, Department of Pharmacology, Faculty of Medicine, Erzincan Binali Yildirim University, Erzincan, Turkey. Conception and design of the study, manuscript preparation.; III Associate Professor, Department of Pharmacology, Faculty of Medicine, Erzincan Binali Yildirim University, Erzincan, Turkey. Conception and design of the study, statistics analysis, manuscript preparation.; IV Associate Professor, Department of Pathology, Faculty of Medicine, Ataturk University, Erzurum, Turkey. Histopathological examinations, manuscript writing.; V Associate Professor, Department of Pharmacology, Faculty of Medicine, Selcuk University, Konya, Turkey. Conception and design of the study, manuscript preparation.; VI Professor, Department of Biochemistry, Faculty of Medicine, Recep Tayyip Erdogan University, Rize, Turkey. Acquisition, analysis and interpretation of data; technical procedures.; VII Assistant Professor, Department of Plant and Animal Breeding, Pazar Vocational College, Recep Tayyip Erdogan University, Rize, Turkey. Conception and design of the study, technical procedures.; VIII Assistant Professor, Department of Internal Medicine, Faculty of Medicine, Erzincan Binali Yildirim University, Erzincan, Turkey. Technical procedures manuscript preparation.; IX Assistant Professor, Department of Chemistry, Faculty of Art end Science, Recep Tayyip Erdogan University, Rize, Turkey. Acquisition, analysis and interpretation of data; technical procedures.; X Professor, Department of Pharmacology, Faculty of Medicine, Erzincan Binali Yildirim University, Erzincan, Turkey. Manuscript writing, critical revision, final approval.

**Keywords:** Adenocarcinoma, Acetates, Usnea, Rats

## Abstract

**Purpose:**

To investigate the effects of the EtOAc extract of *U. longissima* which is uninvestigated previously on esophagogastric cancer induced in rats with N-methyl-N-nitro-N-nitrosoguanidin (MNNG).

**Methods:**

The anticancer activity of EtOAc extract of *U. longissima* was examined in the esophagogastric adenocarcinoma models induced in rats with MNNG. EtOAc extract of *U. longissima,* 50 and 100 mg/kg oral doses were administered once daily for six months. MNNG induced differentiated and undifferentiated type adenocarcinomas in the esophageal and gastric tissues of rats.

**Results:**

EtOAc extract of *U. longissima* obtained from *U. longissima* prevented gastric and esophageal cancerogenesis induced in rats with MNNG. EtOAc extract of *U. longissima* did not have a lethal effect at doses of 500, 1000 and 2000 mg/kg. The prominent anticarcinogenic activity of EtOAc extract of *U. longissima* 50 and 100 mg/kg suggests that it is not toxic and it is selective to the cancer tissue.

**Conclusion:**

This information may shed light on clinical implementation of EtOAc extract of *U. longissima* in future.

## Introduction

 In the treatment of cancer, different methods are used to reduce mortality and morbidity; such as surgery, radiotherapy, chemotherapy, immunotherapy, signal transduction inhibitors, gene therapy and angiogenesis inhibitors^1^. However, according to the recent statistics, more than 8 million people die from cancer every year^2^. This shows that modern medicine lacks effective curative options for the treatment of cancer patients. The current drug therapy does not produce the desired response due to the doses having a toxic effect not only on cancer cells but also on healthy cells^3^. For cancer treatment to be successful, substances that only have a toxic effect on cancer cells are needed. Therefore, there is increasing amount of global efforts to produce synthetic, herbal and fungal drugs to treat various types of cancer^4^ ^,^ ^5^. Research suggests that lichens and their metabolites can be used as an alternative method for treating cancer^3^. Lichens are not a single organism; they are symbiotic, composite organisms composed of fungi (ascomycetes, basidiomycetes) and photosynthetic algae^6^. In this lichen structure, algae and fungi act as single individuals; however, in symbiosis they can produce chemicals that they cannot produce alone. Some of the thousands of secondary metabolites produced by lichens are fulvic, protolichesterinic, fusidic, lobaric, fumarprotocetraric and usnic acids, as well as depsides and depsidons^7^. In this study, the anticarcinogenic activity of a lichen type acid, usnea longissima, was investigated. Although lichens have a medical value due to their antibiotic, antifungal, antiviral and anticarcinogenic properties, only a few of the several types have been researched and reported to exhibit anticarcinogenic activity^8^ ^,^ ^9^. A common problem related to natural drugs is the lack of information about their pharmacologic activities and active components^4^. The anticarcinogenic activities of these chemicals can be investigated through in vivo and in vitro experiments^10^. The commonly used in vivo models involve the induction of gastric and esophageal cancer by MNNG^11^. The literature contains research on the effect of the extracts of different lichen types on gastric and esophageal cancer induced by MNNG. In this study, we investigated the effects of an ethyl acetate (EtOAc) extract of *U. longissima* on esophagogastric cancer (adenocarcinoma) induced by MNNG in rats. The reason for using ethyl acetate extract was found to be more effective than other extracts (water extract, ethanol extract, methanol extract) in cell cultures. However, ethyl acetate are mostly suitable for diffractaic acid, usnic acid and evernic acid wich were the majör compounds of *U. longissima* ^12^.

## Methods

 The study was approved by the university Local Ethical Committee on Animal Experimentation (dated March 3, 2014 and numbered 2014/19).

For the experiment, 64 Albino Wistar male rats weighing 125-137 grams were obtained from the Experimental Application and Research Center of Recep Tayyip Erdoğan University, Turkey. Prior to the experiment in the Pharmacology Department laboratory, the subjects were adapted to the laboratory setting by being kept at room temperature (22°C) for one week with *ad libitum* access to standard laboratory chow and water. 

###  Lichen material 

 The lichen *U. longissima* was collected in 2013 and 2014 from forests near Trabzon and Rize in Turkey. The material was identified and stored in the herbarium of the Faculty of Science and Literature (No: HB 1029). 

###  Chemicals 

 The chemicals used in this study were; MNNG (ABRC, Germany), sodium thiopental (IE Ulagay, Turkey), and cisplatin (Kocak Farma, Turkey). The lichen extract was prepared with ethyl acetate obtained from Sigma. The devices used in the experiments included a centrifuge (Hettich Universal 320 R), an ultrasonic bath (Bandelin Sonorex), and a magnetic stirrer (IKA RCT Basic).

###  Preparation of the lichen extract 

 A 100-gram ground lichen sample was placed in a brown flask and dissolved in 1000 mL ethyl acetate over two hours using an ultrasonic bath. After filtration, the same procedure was repeated for the residue. The filtered extract was then evaporated at 40°C to obtain a dried residue of the crude extract.

###  Experimental groups 

 Forty rats were equally divided into the following four groups (n=10); the healthy control group (HC), the experimental control group (EMC) that only received 200 mg/kg MNNG, the experimental group that received 50 mg/kg EtOAc extract of *U. longissima*+200 mg/kg MNNG (EM-50), and the experimental group that received 100 mg/kg EtOAc extract of *U. longissima*+200 mg/kg MNNG (EM-100).

###  Experimental procedure 

 Gastric and esophageal adenocarcinoma is induced in rats using different doses of MNNG for different durations^13^ ^,^ ^14^. In the current study, we first administered by oral gavage 50 mg/kg and 100 mg/kg EtOAc extract of *U. longissima* to the EM-50 and EM-100 groups, respectively. The HC group was administered by oral gavage the polysorbate-80 solvent. EtOAc extract of *U. longissima* administration was repeated once every days for three months. One hour after the administration of EtOAc extract of *U. longissima*, adenocarcinoma was induced in all the groups except for HC by 200 mg/kg MNNG orally by gavage. MNNG admistration was repeated once every 10 days for three months. In a previous study, MNNG was used at 200 mg/kg döşe for gastric adenocarcinoma model in animals(15). EtOAc extract of *U. longissima* and Tween-80 were also administered at the above-mentioned doses every day over six months. At the end of the six-month period, all the rats were sacrificed with a high dose of anesthesia (sodium thiopental, 50 mg/kg). Macroscopic, histopathological and immunohistochemical examinations were performed on the esophagus and stomach of the subjects. The results obtained from the two EtOAc extract of *U. longissima* groups were compared to those from the control groups. 

###  Histopathological examination 

 For the microscopic examination, four samples were obtained from the esophagogastric junction, antrum, corpus and fundus of each subject. Following the paraffin-embedding process, four-micron-thick sections were cut from each block, stained with eosin hematoxylin, and blindly evaluated by two independent pathologists using a Zeiss Scope A1 light microscope. The findings were graded as follows; grade 0: normal tissue, grade I: hyperplasia in the stratified squamous epithelium, grade II dysplasia in the stratified squamous epithelium, grade III: squamous cell carcinoma, and grade IV: undifferentiated tumors. The changes observed in some preparations could not be classified in microscopic detail and required further immunohistochemical examination.

###  Immunohistochemical examination 

 For the immunohistochemical examination, four-micron-thick sections were fixed on a positively charged slide, and placed in an automated immunohistochemistry device (Leica Bond-Max).

###  Acute toxicity tests of EtOAc extract of U. longissima 

 To test the acute toxicity of EtOAc extract of *U. longissima*, we used 24 rats and for each group 6 rats. three groups of subjects were administered 500 mg/kg EtOAc extract of *U. longissima* (E-500), 1000 mg/kg EtOAc extract of *U. longissima* (E-1000) or 2000 mg/kg EtOAc extract of *U. longissima* (E-2000) by oral gavage. For the healthy control (HC) group, the same volume of distilled water was applied as the solvent. The treated subjects were monitored for 24 hours. In the literature, acute toxicity is evaluated according to the number of animals that die within 24 hours of the treatment (16); therefore, after 24 hours, we took blood samples from the subjects and examined the heart, liver and kidney functions. 

###  Creatinine Kinase-MB (CK-MB) detection 

 Roche/Hitachi cobas c 701system was used to determine whether there was creatinine kinase-MB in the plasma of the subjects. All the steps were performed with the immunologic UV test using the available reagents in the kit according to the recommended procedure.

###  Troponin-I (TP-I) detection 

 The TP-I levels of the plasma of the subjects were measured on a VIDAS Troponin I Ultra kit using the Enzyme-Linked Fluorescent Assay technique. All the steps of the test were automated using the test reagents available in this kit.

###  Detection of alanine aminotransferase (ALT) and aspartate transaminase (AST) 

 Venous blood samples were collected in tubes without anticoagulant. The serum was separated by centrifugation after clotting and stored at 80ºC until assayed. Serum AST and ALT activities, as liver function tests were measured spectrophotometrically in a cobas 8000 (Roche) modular analyzer using commercially available kits (Roche Diagnostics, GmBH, Mannheim, Germany).

###  Creatinine detection 

 The quantitative detection of serum creatinine was spectrophotometrically performed using a Roche cobas 8000 analyzer. This kinetic colorimetric test is based on Jaffe’s method^17^. 

###  Blood urea nitrogen (BUN) detection 

 Serum BUN was quantitatively detected by the spectrophotometry using a Roche cobas 8000 analyzer according to the following formula: BUN = URE* 0.48.

###  Statistical analysis 

 The results of the experiments were presented as ‘mean±standard error of the mean’ (x±SEM). The ANOVA test was used to determine the significance of difference between the groups, followed by the post hoc Tukey-HSD test. Significance of inter-group differences for histopathologic findings was assessed using Kruskal Wallis ANOVA test. Next, Mann-Whitney U was performed. The statistical analyses were performed using ‘IBM SPSS Statistics Version 20’ at a significance level of p<0.05.

## Results

###  Macroscopic findings 

 The macroscopic examination revealed gray-white neoplastic formations of variable sizes (1-3 cm), some being sessile and some with a defined stalk, near the esophagogastric junction only in the EMC group. Only thickening and flattening was observed in the EM-50, EM-100 groups, but no apparent mass lesion was detected.

###  Histopathological findings 

**Figure 1:**
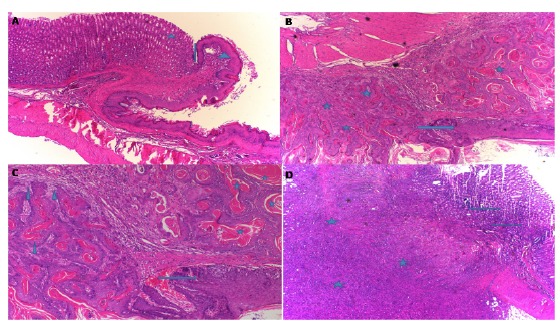
Histopathological appearance of the esophagogastric tissue in the groups. **A.** In HC group, the sections shows esophagogastric junction (*arrow*), mature gastric epithelial cells (*asterisk*) and mature esophageal squamous cells (*arrowhead*) (HE, x100). **B.** In the EMC group, the sections shows well-differentiated mature squamous epithelium (*arrow*) and squamous cell carcinoma (*asterisk*) (HE, x100). **C.** In the EMC group, the sections shows keratin pearls (*asterisk*), invasion (*arrowhead*), and the mature squamous cells (*arrow*) in the well-differentiated squamous cell carcinoma area (HE, x200). **D.** In the EMC group, the sections shows localized tumors (*asterisk*) in the esophageal squamous epithelium and gastric mucosa.

 In the HC group classified as grade-0, the normal histological appearance of the esophagogastric junction (arrow), mature gastric epithelial cells (asterisk) and mature esophageal squamous cells (arrowhead) was observed (Fig. 1A). Figure 1B presents the histopathological results of the EMC group, characterized by well-differentiated mature squamous epithelium (arrow) and squamous cell carcinoma (asterisk). These findings are compatible with grade-III. In the EMC group revealed keratin pearls (asterisk) and invasion (arrowhead) in the well-differentiated squamous cell carcinoma area, and the well-differentiated carcinoma in mature squamous cells (arrow) (Fig. 1C). In addition, there were localized tumors (asterisk) under the esophageal squamous epithelium and gastric mucosa in the EMC group. These tumors were not in keeping with carcinoma foci with classic flat epithelial cells and contained intense mitotic activity and atypical mitosis with large hyperchromatic nuclei, and some had bizarre appearance. The cytoplasmic boundaries were not distinct and there were large atypical cells with cytoplasm (Fig. 1D). 

**Figure 2:**
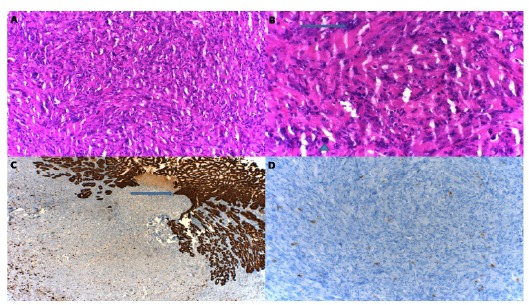
Appearance of the esophagogastric tissue in the EMC group from the immunohistochemistry analysis. **A**; cellularity and atypia tumor cells (HE, x200), **B**; bizarre cells (*arrow*) and mitosis (*arrowhead*) (HE, x400), **C**; normally esophagogastric tissue (*arrow*) (Pan-cytokeratin, x100), **D**; undifferentiated tumors cells (vimentin, x100).

The tissue in this site was evaluated as undifferentiated tumor (Grade-IV). This tissue was further examined by immunohistochemistry, which revealed tumor cells displaying cellularity and atypia (Fig. 2A), bizarre cells (arrow) and mitosis (arrowhead) (Fig. 2B). Pan-cytokeratin x100 did not indicate any reactivity in the tumor cells (Fig. 2C), and non-reactive tumor cells were indicated by vimentin (Fig. 2D).

The tumor cells were also found to be non-reactive to the following; epitelial membrane antigen (EMA), Leucocyte Common Antigen (LCA), anti-alpha Smooth Muscle Actin (SMA), S-100, CD34, C-kit and Myo-D1 which confirmed the classification as undifferentiated tumor sites. 

**Figure 3:**
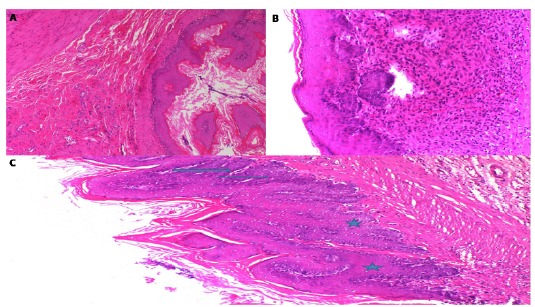
Histopathological appearance of the esophagogastric tissue **A**; in the EM-50 group (HE, x100). There were no pathological findings in the 8 subjects. **B**; in the EM-50 group (HE, x200). Hyperplasia (grade-I) was indicated in the 2 subjects. **C**; in the EM-100 group. The sections shows dysplasia (*arrow*) (HE, x100).

In the EM-50 group, there were no pathological findings in 8 of the 10 subjects (Fig. 3A). In the remaining two, there was an increase in the coefficient of the esophageal epithelium, which indicated hyperplasia (grade-I) (Fig. 3B). In the EM-100 group, dysplasia (arrow) was only observed in the esophagus of two animals (Fig. 3C). 

###  Acute toxicity findings 

 The doses of 500, 1000 and 2000 mg/kg EtOAc extract of *U. longissima* administered rat groups were not observed death. Therefore, the EM50 value of the EtOAc extract of *U. longissima* could not be determined and it was found that therewas no acute lethal effect.

###  CK-MB and TP-I levels 

**Figure 4:**
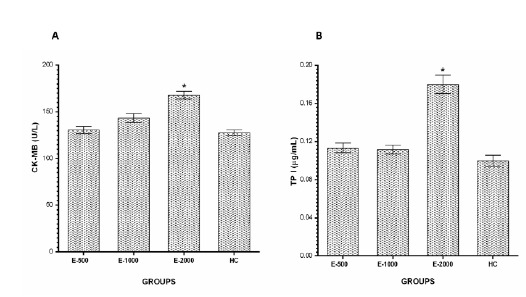
CK-MB (**A**) and TP-I (**B)** levels in the blood samples. E-500, E-1000 and E-2000 groups were compared with the HC group(*P<0.001).

 The levels of CK-MB in the E-500 and E-1000 groups were not significantly increased compared to HC group (p>0.05); however, CK-MB level of E-2000 group was significantly incerased compared to HC group (p<0.001) (Fig. 4A). Similarly, the TP-I level was only found to significantly increase in the E-2000 group compared to the HC group (p<0.001) however, TP-I levels of E-500 and E-1000 groups not significantly increased compared to HC group (p>0.05) (Fig. 4B).

###  ALT and AST levels 

**Figure 5:**
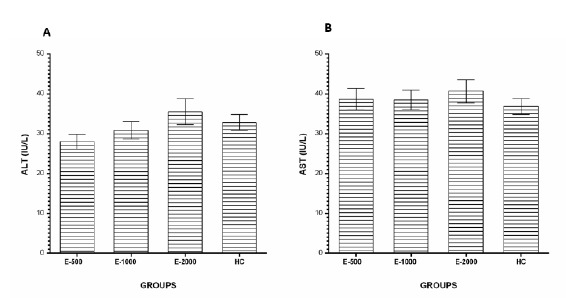
ALT (**A**) and AST (**B**) levels in the blood samples. E-500, E-1000 and E-2000 groups were compared with the HC group.

 No statistically significant difference was found in the E-500, E-1000 and E-2000 groups in terms of serum ALT (Fig. 5A) and AST levels according to HC gropu (p>0.05) (Fig. 5B).

###  BUN and creatinine levels 

**Figure 6:**
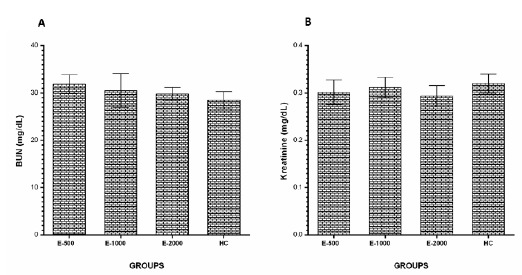
BUN (**A**) and Creatinine (**B**) levels in the blood samples. E-500, E-1000 and E-2000 groups were compared with the HC group.

 The serum BUN (Fig. 6A) and creatinine (Fig. 6B) levels in the E-500, E-1000 and E-2000 groups were similar in the HC groups (p>0.05).

## Discussion

 This study investigated the effect of EtOAc on the esophagogastric adenocarcinoma induced in rats by MNNG. The results of the experiment showed the formation of carcinoma and undifferentiated tumor sites in the esophagogastric tissue of the rats in the EC group. As described in the methodology section, the esophagogastric cancer model was induced by an oral injection of MNNG. The review of the literature shows that MNNG is used in different doses and for different durations to develop a cancer model. For example, Wang *et al.* ^18^ reported that they induced gastrointestinal cancer in rats mixing MNNG with their drinking water for 24 weeks. In an earlier study, we used a similar dose of MNNG over a similar period to induce cancer in rats^15^. Sugano *et al.* ^19^ classified gastric gastric carcinoma into two main groups as differentiated (papillary and tubular adenocarcinoma) and undifferentiated (poorly differentiated signet-ring cell carcinoma). The pathological findings from previous reports and observed in the current study show that we developed an appropriate cancer model to investigate the effects of the chemical of interest on gastric and esophageal carcinoma. 

In this study, hyperplasia was observed in the esophagus of the 2% of rats in the EM-50 group and dysplasia was detected in the 2% of the EM-100 group. When the activity of the EtOAc extract of *U. longissima* doses was compared, 50 mg/kg was found to be more effective than 100 mg/kg. Dysplasia has gained importance in the identification of precancerous lesions in endoscopic biopsies of the stomach and in the differential diagnosis to eliminate invasive carcinoma^20^. There have been various studies concerning the anti-carcinogenic activity of certain lichen types^21^ ^,^ ^22^. The in vitro anticarcinogenic effects of other lichen types, such as *P. caperata*, sulcata and saxatilis on *FemX* (human melanoma) and LS174 (human colon carcinoma) cell lines have been reported^23^. Similarly, Millot et al. demonstrated the cytotoxic activities of several compounds isolated from the lichen Ochrolechia parella such as chloro-depsidone, variolaric acid, lecanoric acid, alpha-alectoronic acid, atranorin and ergosterol peroxide against cancer cells^24^. In another study, the secondary metabolites of sphaerophorin and pannarin isolated from the lichens S*phaerophorus globosus, Psoroma reticulatum, P. pulchrum,* and *P. palladium* were found to be toxic to the cultured human melanoma cells *(*M14 cell line), and it was suggested that this cytotoxic effect resulted from apoptosis induced by the fragmentation of cell DNA(25). Bézivinet *et al.* ^26^ detected the activities of extracts of several lichens (Cladonia convoluta, Cladonia rangiformis, Evernia prunastri, Parmelia caperata, Parmelia perlataPlatismatia glauca, Ramalina cuspidata, and Usnea rubicunda) at different concentrations in DU145, breast and leukemia cell lines. However, to date, no study was found in the literature that investigated the anticarcinogenic activity of Usnea longissima.

As mentioned above, drug therapy does not produce the desired result in the treatment of cancer due to the toxic effect not only on cancer tissue but also on healthy tissue^3^. In the current study, EtOAc extract of *U. longissima* 50 and 100 mg/kg prevented MNNG from inducing esophagogastric carcinoma, and even the 2000 mg/kg dose was not lethal. This high dose (2000 mg/kg) did not even affect serum ALT and AST activities, which are most commonly used to assess liver function^27^. Furthermore, at the same dose, EtOAc extract of *U. longissima* did not change the levels of BUN and creatinine, which indicates that kidney functions were not compromised. An increase in the serum BUN and creatinine levels is considered as indicators of non-renal factors being effective and the presence of extreme damage such as the loss of functional nephrons. On the contrary, 2000 mg/kg EtOAc extract of *U. longissima* was found to slightly increase the CK-MB and cardiac TP-I parameters. This may be due to oxidative stress and the resulting damage to the myocardial membrane^28^.

## Conclusions

 The EtOAc extract of *U.* *longissima* has anticancer activity. EtOAc extract of *U.* *Longissima* was found to have no toxic effect. These results indicate that EtOAc extract of *U.* *Longissima* shows selectivity to cancerous tissue. In addition, EtOAc extract of *U.* *Longissima* is thought to be a therapeutic agent against the formation of esophageal and gastric adenocarcinoma. This results of the study may lead to clinical use of EtOAc extract of *U. Longissima* in the future.
